# The Function of Heat Shock Transcription Factors in Sex Differentiation in *Cynoglossus semilaevis*

**DOI:** 10.3390/ani15101443

**Published:** 2025-05-16

**Authors:** Zhijie Li, Xuexue Sun, Haipeng Yan, Lijun Wang, Xihong Li, Na Wang, Min Wei, Wenteng Xu

**Affiliations:** 1 School of Marine Science and Fisheries, Jiangsu Ocean University, Lianyungang 222005, China; zhijieli2023@163.com; 2 Laboratory for Marine Fisheries Science and Food Production Processes, Qingdao Marine Science and Technology Center, Yellow Sea Fisheries Research Institute, Chinese Academy of Fishery Sciences, Qingdao 266071, China; s1506251002@163.com (X.S.); yanhaipeng265438@gmail.com (H.Y.); wnglikun@163.com (L.W.); lixh@ysfri.ac.cn (X.L.); wangna@ysfri.ac.cn (N.W.)

**Keywords:** Chinese tongue sole, *hsf*, sex differentiation, expression pattern, siRNA knockdown

## Abstract

Chinese tongue sole (*Cynoglossus semilaevis*) is an important marine fish in China. It has sexual dimorphism. The weight and growth rate of female fish are much greater than those of male fish. However, high temperatures can induce sex reversal in genetic female fish (ZW) to phenotypic male fish; thus, identifying the genetic elements involved in temperature perception will provide the molecular basis for sex control. The heat shock transcription factor (*hsf*) is known as an important component of temperature sensing and mediates the heat shock response in fish such as *Danio rerio*; however, its function in *C. semilaevis* is unclear. In this study, five *hsf* genes (*hsf1*, *hsf2*, *hsf4*, *hsf5a*, and *hsf5b*) were identified in tongue sole and found to be expressed in the gonads at different developmental stages, peaking from 7M to 1Y. Gonadal in situ hybridization revealed that *hsf* gene signals were mainly localized in germ cells, e.g., sperm in the testis and all-stage oocytes in the ovary. Upon high-temperature stimulation, the expression of the *hsf* gene in the gonads increased gradually with increasing stimulation time, but different *hsf* genes presented different response patterns. After the RNA interference of *hsf* in the testis and ovarian cell lines, a series of sex-related genes, such as *foxl2* and *dmrt1*, significantly changed. In vivo RNA interference had an effect on the female gonads and mainly affected *neurl3* expression. On the basis of these data, we speculate that *hsf* responds to temperature stimulation and plays an important role in sex differentiation. This study helps elucidate the relationship between temperature sensing and sex differentiation in *C. semilaevis*.

## Simple Summary

As an important economic fish, *Cynoglossus semilaevis* has sexual dimorphism, and the growth difference between males and females is great. Studies have shown that the genotypic females of *Cynoglossus semilaevis* can be transformed into pseudomales at high temperatures. Therefore, we studied the heat shock transcription factor *hsf* gene. In this study, we located five *hsf* genes of *Cynoglossus semilaevis* in gonad tissue and found that they were mainly concentrated in sperm cells and oocytes. Under high-temperature stimulation, with the increase in stimulation time, the expression of the *hsf* gene in the gonad gradually increased, but different *hsf* genes showed different reaction patterns. In vivo siRNA interference had effects on female gonads, mainly affecting the expression of *neurl3*. According to these data, we speculate that *hsf* not only responds to temperature stimuli, but also plays an important role in gender differentiation. This study is helpful to clarify the relationship between the temperature perception and sex differentiation of *Cynoglossus semilaevis*.

## 1. Introduction

The Chinese tongue sole (*Cynoglossus semilaevis*) is a marine fish of significant economic importance in Northeast Asia, with a long history of aquaculture in China [1]. In recent years, its production has gradually expanded, driven by its high commercial value and desirable taste [2]. This species exhibits sexual size dimorphism (SSD), with one-year-old females weighing two to four times more than males [3]. Sex differentiation in fish is often influenced by environmental factors, with temperature being one of the most impactful [4]. Studies have shown that genotypic females can be transformed into pseudomales under high-temperature conditions [5]. This temperature regulation is associated with the activation of the heat shock response.

The heat shock response is characterized by a rapid and marked increase in heat shock proteins (HSPs) when exposed to sublethal temperatures. This cellular protection mechanism is primarily regulated at the transcriptional level. In bacteria, the σ32 factor regulates HSP transcription, while in eukaryotes, heat shock transcription factors (HSFs) perform this role by binding to specific heat shock elements (HSEs) in the promoter regions of target genes [6,7]. The expression of Hsps is regulated by *hsf* at the transcription level. *hsf* is an important transcription regulator, which can recognize and bind the heat shock element HSE in the promoter region of Hsps, thus starting the transcription of Hsps [7]. However, there are significant differences in the number and activity of *hsf* genes across organisms [8]. In insects, such as flies, only a single *hsf* gene exists [9], whereas fish and other vertebrates typically have three or four: *hsf1*, *hsf2*, *hsf4*, and *hsf5*. *hsf3* appears to be specific to birds [10].

*hsf1* is widely expressed in vertebrates and plays a crucial role in preventing heat-induced protein damage and in regulating cellular metabolism. Its activity is modulated by various sensory pathways; for example, PGC-α, a key regulator of energy metabolism, can interact with *hsf1* and inhibit its reactivation ability [11]. While *hsf1* is involved in inducible transcription, *hsf2* is associated with the constitutive transcription of heat shock genes [12,13,14]. First identified in 1998 [15], *hsf2* has been shown to compensate for *hsf1* in the transcription of heat shock genes, although it cannot fully replace it [16]. *hsf2* is a highly unstable protein [17,18] capable of inducing DNA-binding stress, but its role in the acute stress response is limited and depends on changes in the cellular environment and the cell cycle stage [19]. *hsf3* plays an irreplaceable role in the heat stress response of birds, and its unique regulatory mechanism and functional characteristics make it an important target for studying heat tolerance and stress adaptation in avian species [20]. *hsf4* is highly expressed in the lens [19] and is structurally distinct from other HSFs. It lacks the inhibitory trimerization domain (HR-C) [21], and therefore generally exists as a trimer bound to HSEs. Finally, *hsf5* is a key member of the HSF family, with a central role in regulating the expression of HSPs and other stress-related proteins. It is broadly involved in crucial biological processes such as the cellular stress response, protein homeostasis, reproductive development, immune regulation, and aging.

In *C. semilaevis*, the *hsf1*, *hsf2*, *hsf4*, and *hsf5* genes have been identified, with *hsf5* comprising two subtypes: *hsf5a* and *hsf5b*. However, it remains unclear whether *hsf* genes are involved in temperature sensing and whether they influence sex differentiation in this species. Addressing these questions is essential for sex control in *C. semilaevis* breeding programs. In this study, phylogenetic analysis, spatiotemporal expression profiling, tissue localization, RNA interference, and high-temperature treatment were performed. These data provide a solid foundation for understanding the role of *hsf* genes in sex differentiation in *C. semilaevis*.

## 2. Materials and Methods

### 2.1. Ethics Statement

The animal experiment was inspected and approved by the Institutional Animal Care and Use Committee at the Yellow Sea Fisheries Research Institute, CAFS (Approve No.: YSFRI-2022035).

### 2.2. Fish and Tissue Collection

*C. semilaevis* was obtained from the Weizhuo Experimental Base in Tangshan City, Hebei Province (Hebei, China). Tissue samples of 40-day-old, 3-month-old, 7-month-old, 1-year-old, and 1.5-year-old female and male fish, including from the spleen, kidney, heart, liver, intestine, muscle, brain, and gonads, were collected and immediately frozen in liquid nitrogen and stored at −80 °C for long-term storage. The gonads of 1-year-old and 1.5-year-old female and male fish were collected and stored in 4% paraformaldehyde fixative (Shandong, China) to generate sections for in situ hybridization (ISH). Caudal fins were cut and stored in anhydrous ethanol (Shandong, China) for DNA extraction and genetic sex identification.

### 2.3. High-Temperature Breeding Experiment

The 50-day-old seedlings of the same group of *C. semilaevis* were selected for high-temperature experiments at the Weizhuo experimental base in Tangshan City. The experiment was divided into three experimental groups: experimental group 1 (high temperature 6D), experimental group 2 (high temperature 3D) and control group (normal temperature group). Each experimental group had 30 fish, and the variables were strictly controlled. Among them, the control group was cultured in a 25 °C water environment for 6 days; experimental group 1 was cultured in a 30 °C water environment for 6 days; experimental group 2 was cultured in a 25 °C water environment for 3 days; and then the water temperature was slowly increased to 30 °C for 3 days. In the three groups, they were fed once every 2 days and changed water once. At the end of the experiment, the gonads were removed, frozen in liquid nitrogen, and then stored in a refrigerator at −80 °C for subsequent experiments.

### 2.4. Gene Family Identification and Evolutionary Analysis

Basic information on *C. semilaevis* was obtained from the NCBI website (https://www.ncbi.nlm.nih.gov/ (accessed on 13 April 2024)) (Table 1), and a phylogenetic tree of *C. semilaevis* and other species was constructed using MEGA 11.0 software.

### 2.5. Primer Design and Synthesis

The primers used were designed according to the *hsf* gene sequence of *C. semilaevis.* All primers were designed using Primer 5.0 software, and β-actin was used as the internal reference gene. The primers were synthesized by Beijing Ruibo Xingke Biotechnology Co., Ltd. (Beijing, China). The primer sequences are shown in Table 2, and MEGA 11.0 software was used for data analysis.

### 2.6. Genomic DNA Extraction and Genetic Identification

Genomic DNA was extracted using the TIANamp Marine Animals DNA Kit (TianGen Biotech, Beijing, China). The codominant sex-specific marker primer scaffold68-2-F/R, which was used for the genetic sex identification of *C. semilaevis*, was used for PCR and electrophoresis detection. Agarose gel electrophoresis of the PCR products revealed that only one band was observed in the male samples, whereas two bands were observed in the female samples [22].

### 2.7. Total RNA Extraction and cDNA Synthesis

Total RNA was isolated using the use of TRIzol reagent (Invitrogen, Carlsbad, CA, USA). cDNA was synthesized using the TaKaRa PrimeScript^TM^ RT reagent Kit with the gDNA Eraser (Perfect Real Time) Reverse Transcription Kit (TaKaRa, Beijing, China). The integrity, concentration, and quality of the RNA were detected using 1% agarose gel electrophoresis and an ultramicro spectrophotometer (DNA/Protein Analyzer P100+) (Thermo, Waltham, MA, USA).

### 2.8. Real-Time Fluorescent Quantitative PCR of the hsf Gene

qPCR was used to detect the expression level of the *hsf* gene in the gonads of *C. semilaevis* at different developmental stages. qPCR was performed using a 7500 Fast Real-time PCR instrument (Thermo, Shanghai, China). The volume of the reaction mixture was 10.0 μL, including 5.0 μL of THUNDERBIRD^®^ NextSYBR^®^ qPCR Mix (TOYOBO, Osaka, Japan), 1.0 μL of the forward and reverse primers, 2.0 μL of cDNA, and 1.0 μL of ddH_2_O. The primers used in this study are shown in Table 2. The experimental data were analyzed using the 2 ^−ΔΔct^ [23] method to calculate the expression level of each gene. SPSS 26.0 was used for the multiple comparative analysis of the data, and a *p* value less than 0.05 indicated that there was a significant difference between the two groups.

### 2.9. In Situ Hybridization of the hsf Gene

The probe primers ISH-*hsf*-T7 and ISH-*hsf*-sp6 with T7 polymerase and SP6 endonuclease site adapters were designed according to the sequence of the CDS region of the *hsf* gene (Table 1). In situ hybridization (ISH) was performed according to the methods of Chen et al. [24].

According to the instructions of the Roche in vitro transcription kit (Roche, Basel, Switzerland) and the digoxin probe, the target fragment was amplified by a high-fidelity enzyme, and then T7 and SP6 RNA probes labeled (Thermo, Shanghai, China) with digoxin were synthesized by in vitro transcription using T7 and SP6 RNA polymerase.

The gonadal tissue sections of 1 year-old *C. semilaevis* were dewaxed and prehybridized at 70 °C for 4 h, after which they were incubated with hybridization solution containing a probe (final concentration of 0.2 g/mL) at 70 °C overnight. The next day, after washing, the sections were blocked with a blocking solution containing goat serum at room temperature for 4 h and then incubated with an anti-digoxin antibody at 4 °C overnight. On the third day, a BCIP/NBT kit (Roche, Basel, Switzerland) was used for chemical coloration to generate signals, and photographs were taken with a Nikon EClIPSE 80i microscope (Nikon, Tokyo, Japan).

### 2.10. Interference of hsf Mediated by siRNA on Gonadal Cell Lines of C. semilaevis In Vivo and In Vitro

Specific siRNAs and negative controls (NCs) were designed and synthesized by Bioengineering Co., Ltd. (Shanghai, China) (Table 3).

#### 2.10.1. In Vitro siRNA-Mediated Knockdown

The ovarian cells and sperm cells of the semismooth tongue sole gonad cell line established in the laboratory through the separation of gonads were cultured for cell transfection [25].

When the density of the gonadal cells reached 60–80%, cell plating transfection could be carried out. We used the CP Regent transfection kit (RiboBio, Guangzhou, China) to transfect siRNA, negative control (siRNA-NC), and positive control (siRNA-cy3) into the gonadal cells of *C. semilaevis*. A total of 3 μL siRNA was first mixed with 60 μL 1X CP buffer and 5 μL CP regent for 5–10 min, and then added to a 12-well plate. siRNA, negative control (siRNA-NC), and positive control (siRNA-cy3) were repeated 3 times each. After 48 h of transfection, the total RNA of each well was extracted by TRIzol reagent (Ambion, Austin, TX, USA), and then reverse transcription was carried out. The expression levels of *hsf1*, *hsf2*, *hsf4*, *hsf5a*, and *hsf5b* in the gonadal cells of *C. semilaevis* transfected with siRNA were detected by the 10 μL qPCR reaction system.

#### 2.10.2. In Vivo siRNA-Mediated Knockdown

According to the recommendation of the biological company, siRNA and the negative control siRNA-NC reagent were injected into the gonad of 40-day-old *C. semilaevis*, and the concentration was 0.05–0.25 nmol/g, which was slightly modified according to the body size. The micro syringe was soaked in 75% absolute ethanol in advance, autoclaved, and dried before use. These *C. semilaevis* were divided into a negative control group (NC) and an experimental group (average of 20 in each group). After 48 h, gonadal tissue was taken out, frozen with liquid nitrogen, and then stored in a refrigerator at −80 °C. The total RNA of each well was extracted by TRIzol reagent, and then reverse transcription was carried out. The expression changes in *hsf1*, *hsf2*, *hsf4*, *hsf5a*, and *hsf5b* in the gonad tissue of adult *C. semilaevis* transfected with siRNA were detected by the 10 μL qPCR reaction system.

#### 2.10.3. Expression of Other Genes After siRNA-Mediated Knockdown

After the detection of the *hsf* gene knockout, the expression of *sox-9*, *neurl3*, *cyp19a*, *dmrt1*, *tesk1*, and *foxl2*, which are related to sex determination and gonadal development, was detected to explore whether these genes were affected (the primer sequences are shown in Table 2).

## 3. Results

### 3.1. Cloning and Characterization of the hsf Gene

The full-length cDNA of *hsf5a* was obtained from *C. semilaevis*. The protein contained 349 amino acids. The predicted relative molecular weight was 38,041.98 Da, and the isoelectric point was 8.37. The full-length cDNA of *hsf5b* was 1119 bp in length and contained 372 amino acids. The predicted relative molecular weight was 41,375.49 Da, and the isoelectric point was 8.25. The full-length cDNA of *hsf1* was 1551 bp in length, and the protein contained 516 amino acids. The predicted relative molecular weight was 56,458.64 Da, and the isoelectric point was 4.62. The full-length cDNA of *hsf2* was 1629 bp in length, and the protein contained 542 amino acids. The predicted relative molecular weight was 60,646.98 Da, and the isoelectric point was 5.02. The full-length cDNA of *hsf4* was 1290 bp in length, and the protein contained 429 amino acids. The predicted relative molecular weight was 48,108.46 Da, and the isoelectric point was 5.27.

**Figure 1:**
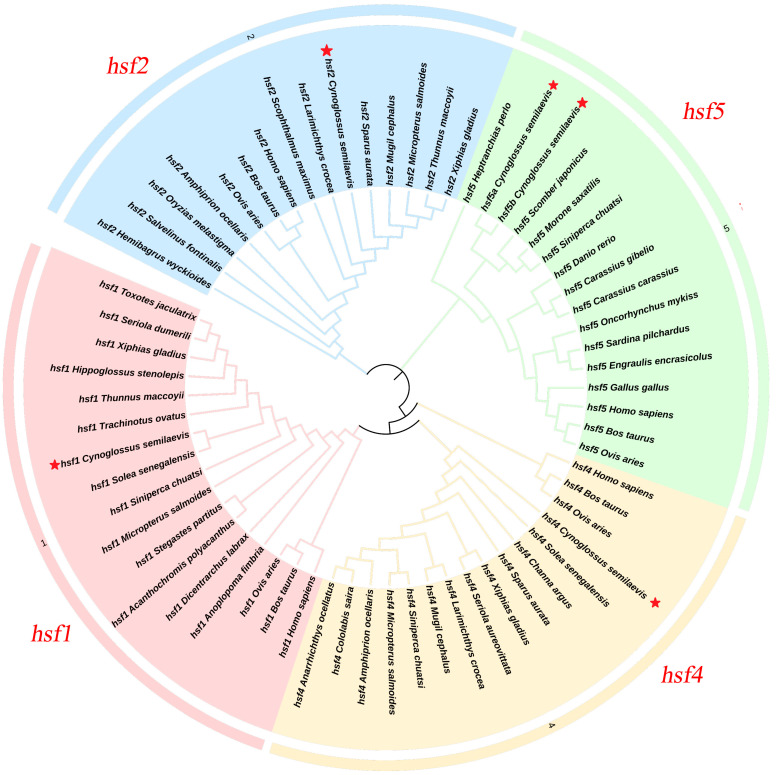
The *hsf1*, *hsf2*, *hsf4*, *hsf5a*, and *hsf5b* sequences of multiple species constitute a phylogenetic tree, and the tongue sole is highlighted in red five stars to show its evolutionary position.

The phylogenetic tree of Figure 1 shows that the *hsf1*, *hsf2*, *hsf4*, *hsf5a*, and *hsf5b* genes of *C. semilaevis* are clustered into a branch with teleosts, but they are far from mammals and birds.

### 3.2. Differential Expression of the hsf Gene in Different Stages of Gonadal Development

**Figure 2:**
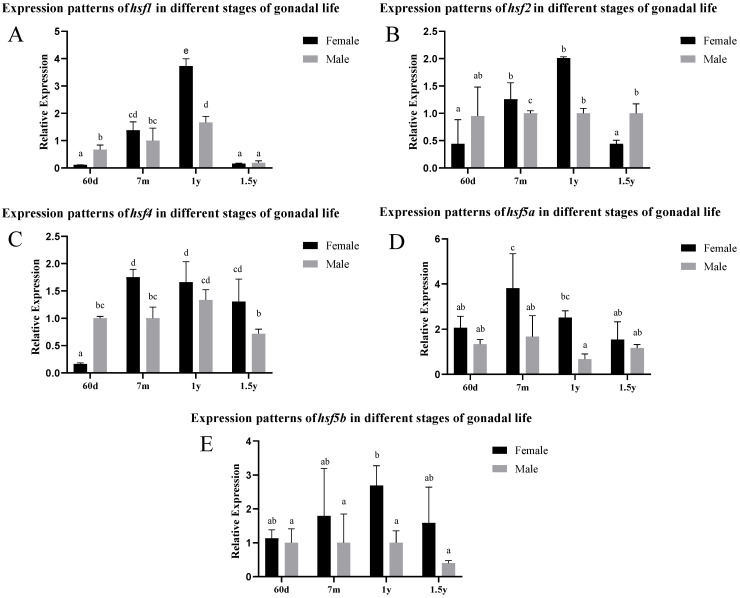
Expression levels of different *hsf* genes in the gonads of *C. semilaevis* at different stages ((**A**): *hsf1*; (**B**): *hsf2*; (**C**): *hsf4*; (**D**): *hsf5a*; (**E**): *hsf5b*). Significant difference (*p* ≤ 0.05). Bars represent mean ± SD from experiments performed in triplicate. Different letters indicate significant differences.

According to the analysis of the gonad expression pattern in Figure 2, the expression levels of *hsf1*, *hsf2*, and *hsf4* in female individuals reached their peak at the age of 1 year, and were significantly higher than those in males from 7 months to 1 year. *hsf5a* and *hsf5b* maintained a high level of expression in female gonads, and the peak values appeared at 7 months and 1 year, respectively. In male individuals, the expression levels of *hsf1*, *hsf2*, and *hsf4* were significantly higher than those of female individuals at 60 days and 1.5 years.

**Figure 3:**
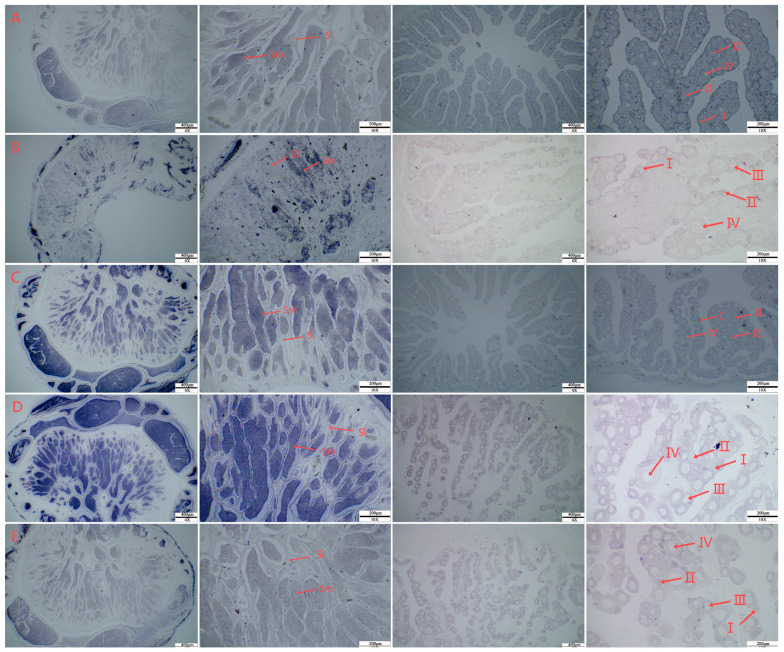
In situ hybridization T7 image of the *C. semilaevis* *hsf* gene ((**A**): *hsf5a*; (**B**): *hsf5b*; (**C**): *hsf1*; (**D**): *hsf2*; (**E**): *hsf4*. I, II, III, and IV were used to mark the different stages of ovarian development. Sm: sperm; Sl: seminal lobule).

In situ hybridization was performed on the gonads of 1-year-old *C. semilaevis*. The signal of the *hsf* gene was mainly localized in sperm cells in the testis, and there was no staining of other tissues, such as sperm lobules. In the ovary, the *hsf* gene signal could be observed in the oocytes at different stages, and the signal was greater in stage III and IV oocytes (Figure 3).

### 3.3. Expression Pattern Analysis of the hsf Gene in the Gonads After High-Temperature Treatment

**Figure 4:**
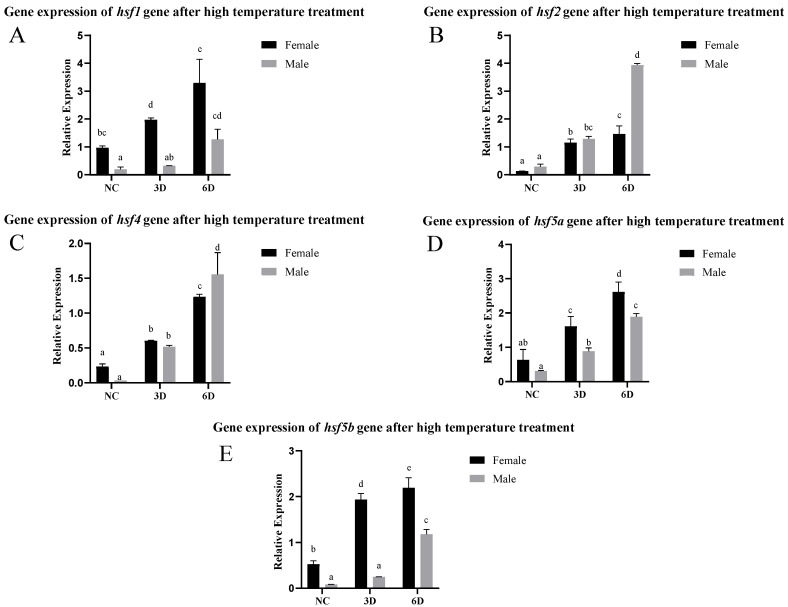
Expression of the *hsf* gene in *C. semilaevis* after high-temperature treatment ((**A**): *hsf1*; (**B**): *hsf2*; (**C**): *hsf4*; (**D**): *hsf5a*; (**E**): *hsf5b*). Significant difference (*p* ≤ 0.05). Bars represent mean ± SD from experiments performed in triplicate. Different letters indicate significant differences.

After high-temperature stimulation, the expression of the *hsf* gene increased significantly, and the longer the stimulation time, the greater the increase in expression. Among these genes, *hsf1*, *hsf5a*, and *hsf5b* were highly expressed in female fish in the NC group, and the expression level in female fish was also significantly greater than that in male fish after stimulation. There was no significant difference in *hsf2* or *hsf4* expression between male and female fish in the NC and 3d treatment groups, and the expression level in male fish was greater than that in female fish after 6d of stimulation (Figure 4).

### 3.4. Analysis of Expression Patterns After siRNA-Mediated Knockout In Vitro and In Vivo

**Figure 5:**
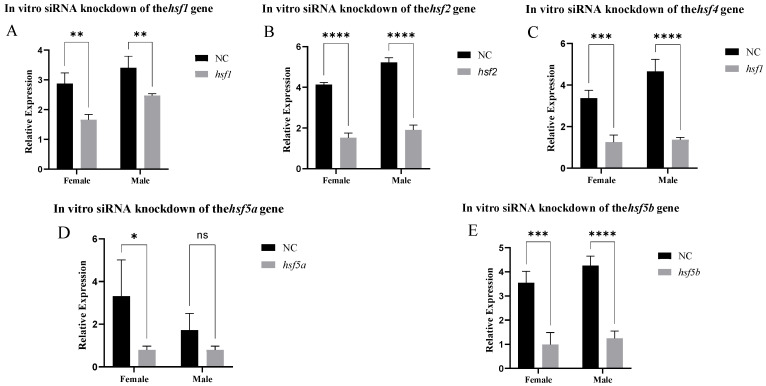
Expression of the *hsf* gene in *C. semilaevis* after knockdown in vitro ((**A**): *hsf1*; (**B**): *hsf2*; (**C**): *hsf4*; (**D**): *hsf5a*; (**E**): *hsf5b*). Significant difference (*p* ≤ 0.05). Bars represent mean ± SD from experiments performed in triplicate. Significant difference (ns: *p* > 0.05, *: *p* ≤ 0.05, **: *p* ≤ 0.005, ***: *p* ≤ 0.0005, ****: *p* ≤ 0.0001).

After siRNA knockout, the *hsf1*, *hsf2*, *hsf4*, and *hsf5b* genes had a good interference effect in the gonadal cells. The *hsf5a* gene only had a good infection interference effect in the ovarian cells (Figure 5). The fluorescence results of positive control siRNA-Cy3 showed that the transfection effect was good (Supplementary Figure S1), and the experimental results were reliable.

**Figure 6:**
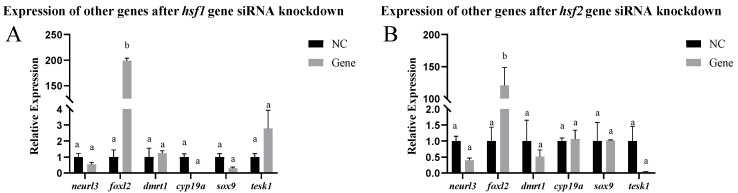

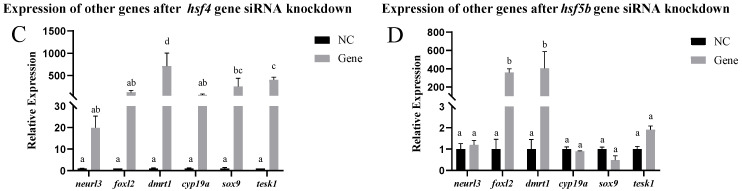
Expression of other genes in the testis cells of the *C. semilaevis hsf* gene after siRNA-mediated knockdown ((**A**): *hsf1*; (**B**): *hsf2*; (**C**): *hsf4*; (**D**): *hsf5b*). Significant difference (*p* ≤ 0.05). Bars represent mean ± SD from experiments performed in triplicate. Different letters indicate significant differences.

In vitro, other genes related to sex differentiation and development were detected in testis cells after siRNA-mediated knockdown of the *hsf1*, *hsf2*, *hsf4*, and *hsf5b* genes. After *hsf1* siRNA-mediated knockdown, the expression of the *foxl2* gene significantly increased, and the expression of the other genes did not change significantly. After *hsf2* siRNA-mediated knockdown, the expression of the *foxl2* gene significantly increased, whereas the expression of the other genes did not change significantly. After knocking down the *hsf4* gene using siRNA, the expression of the *dmrt1*, *sox-9*, and *tesk1* genes increased significantly, whereas the expression of the other genes did not change significantly. After *hsf5b* siRNA-mediated knockdown, the expression of the *dmrt1* and *foxl2* genes increased significantly, whereas the expression of the other genes did not change significantly (Figure 6).

**Figure 7:**
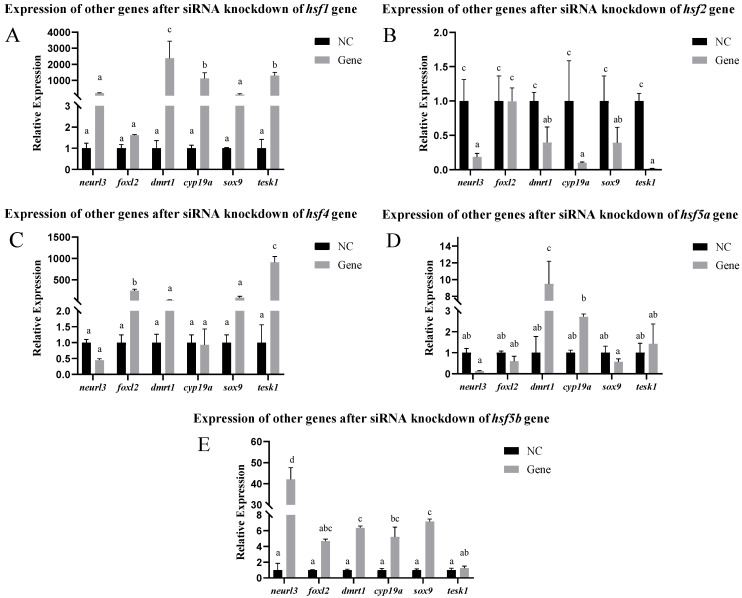
Expression of other genes in *C. semilaevis hsf* gene ovarian cells after siRNA-mediated knockdown ((**A**): *hsf1*; (**B**): *hsf2*; (**C**): *hsf4*; (**D**): *hsf5a*; (**E**): *hsf5b*). Significant difference (*p* ≤ 0.05). Bars represent mean ± SD from experiments performed in triplicate. Different letters indicate significant differences.

After siRNA-mediated knockdown of the *hsf* gene in the ovarian cells, the expression of other related genes changed. After the knockdown of the *hsf1* gene using siRNA, the expression of the *cyp19a*, *dmrt1*, and *tesk1* genes increased significantly, whereas the expression of the other genes did not change significantly. After the knockdown of the *hsf2* gene using siRNA, the expression of the *sox-9, neurl3*, *cyp19a*, *dmrt1*, and *tesk1* genes decreased significantly, whereas the expression of the *foxl2* gene did not change significantly. After knocking down the *hsf4* gene using siRNA, the expression of the *tesk1* and *foxl2* genes increased significantly, whereas the expression of the other genes did not change significantly. After knocking down the *hsf5a* gene using siRNA, the expression of the *dmrt1* gene increased significantly, whereas the expression of the other genes did not change significantly. After *hsf5b* siRNA-mediated knockdown, the expression levels of the *sox-9*, *neurl3*, *cyp19a*, and *dmrt1* genes significantly increased, whereas the expression levels of the other genes did not significantly change (Figure 7).

**Figure 8:**
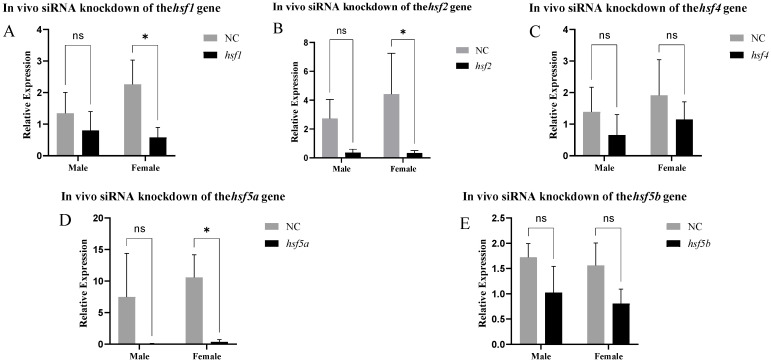
Expression of the *hsf* gene in *C. semilaevis* after knockdown ((**A**): *hsf1*; (**B**): *hsf2*; (**C**): *hsf4*; (**D**): *hsf5a*; (**E**): *hsf5b*). Significant difference (*p* ≤ 0.05). Bars represent mean ± SD from experiments performed in triplicate. (ns: *p* > 0.05, *: *p* ≤ 0.05).

**Figure 9:**
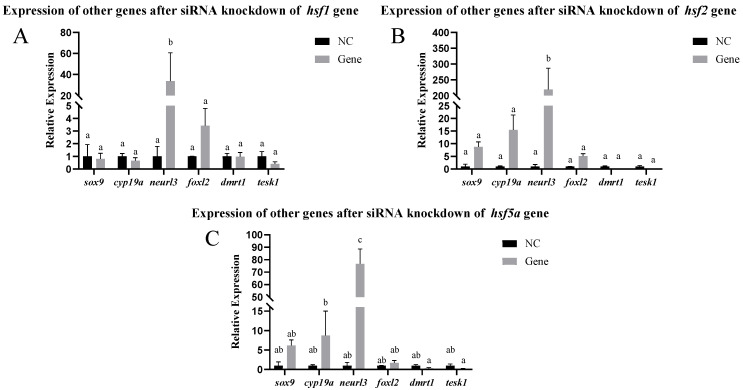
Expression levels of other genes in *C. semilaevis*, namely after the knockdown of ovarian siRNA in vivo ((**A**): *hsf1*; (**B**): *hsf2*; (**C**): *hsf5a*). Significant difference (*p* ≤ 0.05). Bars represent mean ± SD from experiments performed in triplicate. Different letters indicate significant differences.

The results of siRNA knockdown in vivo are shown in Figure 8. Approximately 48 h after siRNA injection, the *hsf1*, *hsf2*, and *hsf5a* genes were significantly knocked down in the ovary. We selected ovaries with *hsf1*, *hsf2*, and *hsf5a* knocked down to detect other sex differentiation- and development-related genes and found that the expression of the *neurl3* gene was significantly increased, whereas that of other genes did not change significantly (Figure 9).

## 4. Discussion

Analysis of the genes from the HSF (Heat Shock Factor) family in the flounder *C. semilaevis* reveal remarkable structural and functional diversity. Five genes are identified—*hsf1*, *hsf2*, *hsf4*, *hsf5a*, and *hsf5b*—whose cDNAs vary between 1050 and 1629 base pairs. The proteins encoded by these genes have molecular weights ranging from 38.0 to 60.6 kDa and isoelectric points from 4.62 to 8.37. The *hsf1* gene, the largest in the family, has 1551 bp, encodes a protein of 516 amino acids, and has the most acidic isoelectric point (pI 4.62), while *hsf5a*, the smallest, has 1119 bp, encodes a protein of 372 amino acids, and has the most basic isoelectric point (pI 8.37).

These variations reflect the wide range of functions performed by these transcription factors, which, throughout evolution, have undergone gene duplications and structural modifications, resulting in different functional domains, expression patterns, and mechanisms of action. Such characteristics allow *hsfs* to regulate not only the response to thermal and environmental stress, but also fundamental processes such as development, cellular homeostasis, and physiological adaptation. In plants, for example, they are involved in the response to drought, salinity, and heat, while in animals, they act on protein stability and cell cycle control [26,27,28,29].

Phylogenetic analyses show that *hsf1*, *hsf2*, and *hsf4* cluster with orthologs from other teleost fishes, while *hsf5a* and *hsf5b* form distinct branches with species such as *Japanese mackerel*. These findings corroborate the knowledge that hsf genes play essential roles in cellular homeostasis in a wide range of species, but with particularities that may be adaptive to the environment and the type of organism. Furthermore, these data suggest that the gene duplications characteristic of teleosts contributed to the functional diversification of these genes, strengthening the idea that their evolution was shaped by environmental pressures and plays an essential adaptive role in the stress response in fish [30,31,32].

The expression results suggest a dynamic pattern in the expression of *hsf* family genes throughout the development of the gonads of *C. semilaevis*, with significant variations between sexes and over time. The expression of the *hsf1*, *hsf2*, *hsf4*, and *hsf5* genes is observed at different stages of development, with peaks of expression at 60 days, 7 months, and 1 year of age, indicating a crucial role of these genes in the regulation of gonadal development and in the response to heat stress.

*hsf1*, widely recognized as a marker of heat stress, shows a significant positive correlation between its expression and the time of heat exposure. This corroborates previous studies conducted in other species, such as *Penaeus monodon* and *Danio rerio*, where increased *hsf1* expression was associated with adaptive responses to thermal variations. In *C. semilaevis*, *hsf1* activation may be a cellular defense mechanism, crucial for the protection of the gonads during heat stress. The differentiation in *hsf1* expression between males and females suggests an adaptive response that may be regulated by sex-specific mechanisms, possibly related to the gametogenesis process [33,34,35].

The expression of the *hsf2* and *hsf4* genes, although important in physiological processes, such as the formation of germ cells and embryonic development, does not present variations which are significant between the sexes in the NC and 3d treatment groups. This may suggest a more stable regulation of these genes during gonadal development and a lower dependence on heat stress in the modulation of the expression of these genes, unlike *hsf1*, which appears to be more sensitive to environmental stress [36].

The genes *hsf5a* and *hsf5b*, which are little-studied in fish, show a particularly high expression in female fish and are highly expressed after heat exposure. This significant increase in expression in females suggests a relevant role of these genes in the response to heat stress and possibly in gametogenesis, especially in female meiosis. The fact that *hsf5* is essential for male meiosis in other species, such as *Danio rerio*, reinforces the hypothesis that this gene also plays a crucial role in the reproduction of *C. semilaevis* [37].

In situ hybridization analysis reveals that the *hsf* gene is predominantly localized in spermatogenic cells in the testes and in oocytes in the ovary, with the most intense signals observed in stages III and IV of oocyte development. These findings suggest that hsf genes play a direct role in gametogenesis, possibly regulating gamete formation and maturation under normal and stress conditions. Studies have shown that in the testes, *hsfs* are present in the early stages of spermatogenesis, being crucial for sperm maturation. In the ovaries, their expression is predominant in primary oocytes and in the early stages of oocyte development, directly influencing female maturation and fertility. Furthermore, studies reveal that these transcription factors are essential for gamete progression under normal and heat stress conditions, controlling processes such as apoptosis and gamete quality. Cooperation between *hsf1* and *hsf2* is vital for fertility in both sexes, with inactivation of these genes resulting in failures in gametogenesis and infertility [38].

The expression of the *hsf* gene increases significantly after heat stress. Notably, *hsf1*, *hsf5a*, and *hsf5b* exhibit similar response patterns in males and females. Among them, the expression levels of *hsf1*, *hsf5a*, and *hsf5b* in female fish are significantly higher than those in male fish in the normal control group (NC group), and after high-temperature stimulation, the expression levels of these genes in female fish further increase significantly, and are generally higher than those in male fish. In the experiment of *Danio Rerio*, *hsf1* and *hsf5* can maintain meiosis and embryo development by directly or indirectly regulating the target gene, and gene knockout reduces the embryo survival rate [39]. This result indicates that *hsf1*, *hsf5a*, and *hsf5b* may play a more important role in the high-temperature stress response of female fish, and perhaps the expression of these genes is regulated by female-specific regulatory mechanisms.

This study systematically investigates the role of the *hsf* gene family in reproductive regulation in *C. semilaevis*. In vitro experiments show that the mRNA levels of *hsf1*, *hsf2*, *hsf4*, and *hsf5b* decrease after silencing the corresponding genes with siRNA. Among them, *hsf5a* shows tissue-specific differences: the knockout efficiency is higher in ovarian cells than in testicular cells. This specificity may be related to sex-specific regulatory elements in the gene’s promoter region, suggesting distinct biological roles in male and female reproductive systems.

It is also found that *foxl2* gene expression is upregulated in testicular cells after the co-knockout of the four *hsf* genes. As a central factor in ovarian differentiation, the abnormal overexpression of *foxl2* may interfere with normal germ cell development. As a member of the FOX gene family, *foxl2* plays a vital role in various developmental processes, including ovarian differentiation and cell specialization [40]. These results suggest that *hsf1*, *hsf2*, *hsf4*, and *hsf5b* may be closely related to ovarian development in *C. semilaevis*.

In vivo experiments show that only *hsf1*, *hsf2*, and *hsf5a* have significant knockout effects in the ovary. Following knockout, *neurl3* expression increases substantially (a phenomenon also observed in ovarian cell lines), and the key spermatogenesis-related gene neur1 may regulate spermatocyte division via ubiquitination [41]. Therefore, this study proposes that *hsf* genes may play dual roles in gamete formation through the *neur1–neurl3* regulatory axis: maintaining oocyte homeostasis in females and mediating the ubiquitination pathway in spermatogenesis in males.

In conclusion, this study explores the dual functions of the *hsf* gene family in reproductive regulation in *C. semilaevis* and reveals that the *hsf* signaling pathway may serve as a bridge connecting male and female reproductive development. These findings provide a theoretical foundation for the development of sex-control technologies in aquatic animals and open new avenues for studying reproductive evolution in vertebrates. Currently, the preliminary data only offer a starting point for understanding the regulatory relationship between *hsf* genes and sex differentiation, and further experiments—such as CRISPR/Cas9—are required. Future plans include single-cell sequencing and chromatin conformation analysis to explore the three-dimensional regulatory networks of these genes during germ cell differentiation.

## 5. Conclusions

In this study, the expression and function of the *hsf* gene in female and male *C. semilaevis* were explored by qPCR, in situ hybridization, and siRNA interference. The results showed that the *hsf* gene was expressed in all stages of the gonad of *C. semilaevis*, and reached its peak from 7M to 1Y. By in situ hybridization, it was found that the *hsf* gene signal in the gonad of *C. semilaevis* was mainly located in the sperm cells in the testis and the oocytes in the ovary, and it was expressed in the oocytes at all stages. When *C. semilaevis* was continuously stimulated by high temperature, it was found that the expression of the *hsf* gene in gonad increased, which was proportional to the time of high-temperature stimulation, indicating that the heat shock response was active with high-temperature stimulation. After siRNA interference in the testis cell line, it was found that the expression levels of *sox-9*, *neurl3*, *cyp19a*, *dmrt1*, *tesk1*, and *foxl2* all changed, and the expression levels of some genes changed significantly. After siRNA interference in ovarian cell lines, it was found that the expression levels of *sox-9*, *neurl3*, *cyp19a*, *dmrt1*, and *foxl2* all changed, and the expression levels of some genes changed significantly. It is speculated that *hsf* may play an important role in the sex differentiation and growth of *C. semilaevis*. This laid a foundation for us to explore the function of the *hsf* gene in the sex differentiation of *C. semilaevis*.

## Figures and Tables

**Table 1:** Overview of *hsf* gene in *C. semilaevis* (NCBI website).

Gene	ID	Chromosome Position	Gene Full Length (bp)	Accession Number		CDS (bp)	Protein Length (aa)
				mRNA	Protein		
*hsf1*	103393760	18	4558	XM_008330832.3	XP_008329054.1	1551	516
				XM_008330833.3	XP_008329055.1	1500	499
*hsf2*	103380605	7	15,272	XM_008312623.3	XP_008310845.1	1629	542
*hsf4*	103379546	6	10,718	XM_008311129.3	XP_008309351.1	1290	429
				XM_017032938.2	XP_016888427.1	1290	429
*hsf5a* (LOC103395174)	103395174	19	4420	XM_008332788.3	XP_008331010.1	1050	349
				XM_008332789.3	XP_008331011.1	1050	349
				XM_017041700.2	XP_016897189.1	1050	349
*hsf5b* (LOC103395175)	103395175	19	4567	XM_025065908.1	XP_024921676.1	1119	372
				XM_017041699.2	XP_016897188.1	504	167
				XM_008332790.3	XP_008331012.1	1146	381

**Table 2:** The sequence of primers.

Primer	Sequence (5′~3′)
q*hsf1*-F	ATCGACTCCAGGATCAACGC
q*hsf1*-R	ATATTTGGGCACGGAGTGGG
q*hsf2*-F	GACTGAGAACGGAGCGATTT
q*hsf2*-R	GTTTGCCGGTTTGCATCATT
q*hsf4*-F	CTCCAGTCCTGCTTCAAAGT
q*hsf4*-R	GCCTTTGTCTCTGGTGTCA
q*hsf5a*-F	AACTCAGCGCTGGTCTCAAA
q*hsf5a*-R	GTCGCAGCATGAGCTGTTTT
q*hsf5b*-F	TTGATGCCACCTCCAACCAA
q*hsf5b*-R	CTTGAATCAAAGCGAGCGGG
β-actin-F	TTCCAGCCTTCCTTCCTT
β-actin-R	TACCTCCAGACAGCACAG
ISH-*hsf1*-sp6	AAGGGGAAACAGGAAACCA
ISH-*hsf1*-T7	GGACCAGAGACACCAGGAA
ISH-*hsf2*-sp6	AGGGATGGTCCTGTGGAGTT
ISH-*hsf2*-T7	TGGGCATCTTTCCATCGTCC
ISH-*hsf4*-sp6	AGAGGGGGAGTAGGAAAGG
ISH-*hsf4*-T7	ATGTATGGTCGGAGGTTGG
ISH-*hsf5a*-sp6	TTTCTGCAGCTTTGTGCGTC
ISH-*hsf5a*-T7	CCAGACAGTTGGGAGTCGTC
ISH-*hsf5b*-sp6	ATCTTGTCTCCTTCCCCCAG
ISH-*hsf5b*-T7	GTTTTTGCATTCGCCACTTC
*Sox-9*-qF	AAGAACCACACAGATCAAGACAGA
*Sox-9*-qR	TAGTCATACTGTGCTCTGGTGATG
*cyp19a*-qF	GGTGAGGATGTGACCCAGTGT
*cyp19a*-qR	ACGGGCTGAAATCGCAAG
*neurl3*-qF	CTGGTGTTTAGCAGCCGTCCT
*neurl3*-qR	CCAGAACTCCAGCACTGACCC
*foxl2*-qF	GAGAGGAAGGGCAACTACTGGA
*foxl2*-qR	TGGTTGGAAGTGCGTGGG
*dmrt1*-qF	GGAGGAAGAACTTGGGATTTG
*dmrt1*-qR	AGGTAGGAGGTTGCTGGG
*tesk1*-qF	GCAGAAACTCTCTCACCCCAACA
*tesk1*-qR	CCAGACCAAAGTCCGTCACCA

**Table 3:** Sequence of siRNA primer of *C. semilaevis hsf* gene.

Primer	Sequence (5′~3′)
si-*hsf1*-F	AAGAAGUUCUGCCAAAGUATT
si-*hsf1*-R	UACUUUGGCAGAACUUCUUTT
si-*hsf2*-F	AUGGAAAGAUGCCCAAAUGTT
si-*hsf2*-R	CAUUUGGGCAUCUUUCCAUTT
si-*hsf4*-F	AAGAAGUUCUUCCAAAGUATT
si-*hsf4*-R	UACUUUGGAAGAACUUCUUTT
si-*hsf5a*-F	GGCACCUUAUCGAGUAACATT
si-*hsf5a*-R	UGUUACUCGAUAAGGUGCCTT
si-*hsf5b*-F	UGCUGAUAACAAGGCUAAATT
si-*hsf5b*-R	UUUAGCCUUGUUAUCAGCATT

## Data Availability

The data presented in this study are available in the article.

## Supplementary Materials

The following supporting information can be downloaded at: https://www.mdpi.com/article/10.3390/ani15101443/s1, Figure S1: Transfection efficiency of *C. semilaevis* cells. A and B are testis cells, and C and D are ovarian cells; A and C are cell states under normal light, and B and D are cell states under red fluorescence.
