# Supplementary material for: The Function of Heat Shock Transcription Factors in Sex Differentiation in Cynoglossus semilaevis
Source: Animals (Basel). 2025 May 16;15(10):1443. doi: 10.3390/ani15101443 (PMC12108280; doi:10.3390/ani15101443)
Supplement: Supplementary file 1 [file animals-15-01443-s001.zip › animals-3565474-supplementary.pdf]

Supplementary Figure S1

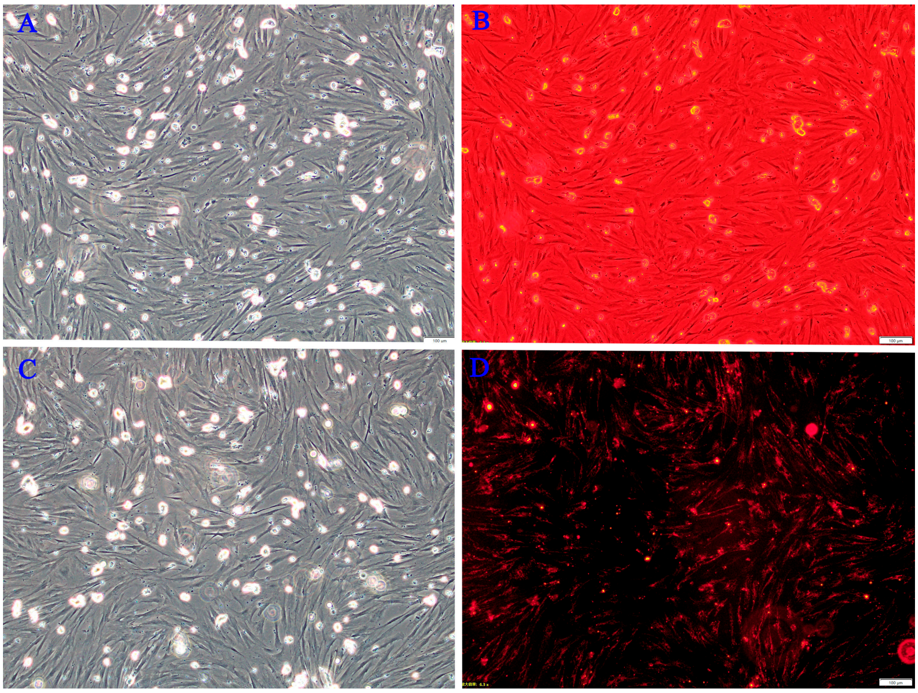

Figure S1 Transfection efficiency of C. semilaevis cells. A and B are testis cells, and C and D are ovarian cells; A and C are cell states under normal light, and B and D are cell states under red fluorescence
